# Alumina-Doped Silica Aerogels for High-Temperature Thermal Insulation

**DOI:** 10.3390/gels7030122

**Published:** 2021-08-14

**Authors:** Yu Wu, Xiaodong Wang, Lin Liu, Ze Zhang, Jun Shen

**Affiliations:** Shanghai Key Laboratory of Special Artificial Microstructure Materials and Technology, School of Physics Science and Engineering, Tongji University, Shanghai 200092, China; 1410560@tongji.edu.cn (Y.W.); 1930976@tongji.edu.cn (L.L.); zzzhangze@163.com (Z.Z.)

**Keywords:** silica aerogels, alumina-doped, high temperature resistance, thermal stability

## Abstract

In this study, we used two methods to prepare alumina-doped silica aerogels with the aim of increasing the thermal stability of silica aerogels. The first method was physical doping of α-Al_2_O_3_ nano powders, and the second method was to create a chemical compound via the co-precursor of TEOS and AlCl_3_·6H_2_O in different proportions. The shrinkage, chemical composition, and specific surface area (SSA) of samples after heating at different temperatures were analyzed. Our results show that the silicon hydroxyl groups of samples derived from AlCl_3_·6H_2_O gradually decreased and nearly disappeared after heating at 800 °C, which indicates the complete dehydration of the silicon hydroxyl. Thus, the samples exhibited a large linear shrinkage and decreased SSA after high-temperature heat treatment. By contrast, samples doped with α-Al_2_O_3_ powders retained abundant silicon hydroxyl groups, and the 6.1 wt.% α-Al_2_O_3_-doped sample exhibited the lowest linear shrinkage of 11% and the highest SSA of 1056 m^2^/g after heat treatment at 800 °C. The alumina-doped silica aerogels prepared using a simple and low-price synthesized method pave the way for the low-cost and large-scale production of high-temperature thermal insulation.

## 1. Introduction

Silica aerogels are considered important and useful materials due to their nanoporous structure, ultra-low density, low thermal conductivity, high porosity and high specific surface area. Following about 90 years of development, the production of silica aerogels has been industrialized, and they have been widely applied, especially in the thermal insulation field [1,2,3,4]. Over recent years, research on silica aerogels and their composites has focused on synthesis parameters [5,6], multivariate composites [7,8], mechanical reinforcement [9,10] and industrial applications [11,12]. Different silica aerogel composite materials, such as blankets, paint, coatings and cloth, have been applied in buildings, oil pipelines and as industry furnace insulation. However, pure silica aerogels sinter at temperatures above 600 °C, which limits their high-temperature applications.

Metal oxides, such as Al_2_O_3_ [13,14,15,16,17,18], ZrO_2_ [19,20,21,22] and TiO_2_ [23,24,25,26]) aerogels, possess better thermal stability and thermal insulation properties than silica aerogels at temperatures above 600 °C, especially alumina aerogels. For example, Zu et al. [27] prepared silica-doped alumina aerogels with high heat resistance, which showed a partial θ-Al_2_O_3_ phase and a high SSA of 136 m^2^/g after heat treatment at 1300 °C. However, the related research remains in the laboratory stage and depends on expensive raw materials and complex processes, making it difficult to carry out large-scale industrial production. Fabricating silica-based aerogels with enhanced heat resistance using an inexpensive process is critical to the development of industrial high-temperature applications. Preparing a silica–alumina composite aerogel represents a potentially effective method for enhancing the heat resistance of aerogels, but the specific synthesis methods (i.e., precursors, composite modes and sol-gel parameters) need to be studied. Currently, the precursors of alumina for aerogels mainly include aluminum alkoxides, such as aluminum sec-butoxide (ASB) or isopropoxide (AIP), and alumina inorganic salts such as aluminum chloride (AlCl_3_·6H_2_O) and aluminum nitrate (Al(NO_3_)_3_·9H_2_O). Although aluminum alkoxides are the main precursors used to fabricate alumina or alumina composite aerogels, they have some defects that render them unsuitable for industrial production. Aluminum alkoxides have ultra-high reactivity and complex chemical pathways; thus, the reaction process is difficult to control. Moreover, aluminum alkoxides are relatively expensive and are not environmentally friendly. By contrast, alumina inorganic salts are easily controlled during gel preparation and are more economical. Many strategies, including ion-exchange resin, electrosorption ion removal and inorganic ion exchanger [28,29,30,31] for chloride ion removal, have been developed. Wu et al. [32] fabricated Al_2_O_3_-SiO_2_ composite aerogels via TEOS and AlCl_3_·6H_2_O as precursors, which showed a γ-Al_2_O_3_ phase and high SSA of 630.6 m^2^/g after heat treatment at 600 °C. However, the SSA of Al_2_O_3_-SiO_2_ composite aerogels decreased to 277.7 m^2^/g due to volume shrinkage after heat treatment at 1000 °C. Chen et al. [33] prepared Al_2_O_3_-SiO_2_ aerogels using TEOS and AlCl_3_·6H_2_O as precursors and found that the samples with a molar ratio of Al/Si = 3:1 possessed better thermal insulation performance and thermal stability, but the alumina proportion was ultra-high, which was not suitable for industrial-scale production. Lei et al. [34] improved the thermal insulation of silica aerogels doped with nano-size Al_2_O_3_ powders. They found that the macropore volume fraction was dramatically reduced from 63.05% to 23.12%, and that the SSA decreased from 837.4 m^2^/g to 358.5 m^2^/g with the addition of Al_2_O_3_ powders at room temperature; however, they did not mention its high-temperature properties. As using alumina inorganic salts is preferable for industrial production, we selected AlCl_3_·6H_2_O and α-Al_2_O_3_ powders as the raw materials in low proportions to improve the high-temperature performance of silica aerogels and examined the feasibility of this method for large-scale production.

In this paper, we present an acid-base, two-step sol-gel method for the synthesis of monolithic alumina-doped silica aerogels using AlCl_3_·6H_2_O and α-Al_2_O_3_ powders as the raw materials. We applied two composite methods: Physical doping of α-Al_2_O_3_ nano powders and creating a chemical compound through the co-precursor of tetraethyl orthosilicate (TEOS) and AlCl_3_∙6H_2_O in low proportions. These methods are low-cost and environmentally friendly as they lack alumina alkoxides and epoxide addition. Furthermore, these methods may promote the industrialization of silica-based aerogels in the application of high-temperature (600–1000 °C) thermal insulation.

## 2. Results and Discussion

### 2.1. Macroscopic Properties of As-Prepared Samples

**Figure 1:**
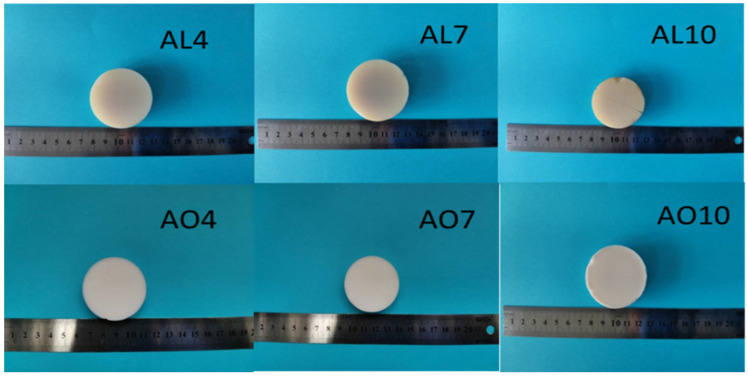
Photos of alumina-doped silica aerogels formed with different precursors and proportions without heat treatment (AL and AO represent the samples derived from AlCl_3_·6H_2_O and α-Al_2_O_3_ powders, respectively. The numbers denote the molar percentage of Al/(Al+Si)).

Figure 1 shows the macro-morphology of the as-prepared samples. After supercritical drying, both methods obtained semitransparent, monolithic and crack-free alumina-doped silica aerogels. The AL samples exhibited lower density and thermal conductivity than the AO samples with the same molar percentage (Table 1), and AL4 had the lowest thermal conductivity of 0.0269 W/m·K.

**Figure 2:**
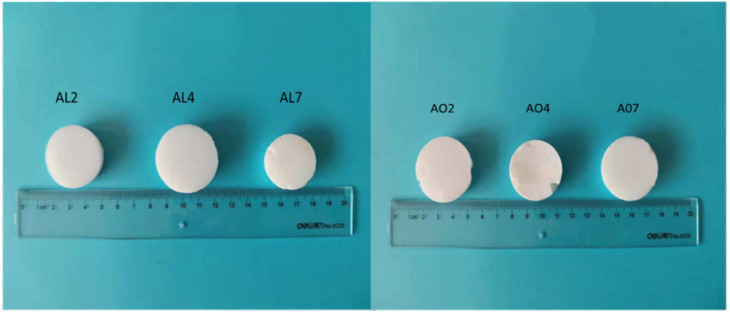
Photos of alumina-doped silica aerogels after heat treatment at 1000 °C (AL and AO represent the samples derived from AlCl_3_·6H_2_O and α-Al_2_O_3_ powders, respectively. The numbers denote the molar percentage of Al/(Al+Si)).

After heat treatment at 1000 °C for 1 h, most samples retained a monolithic and crack-free macro-morphology as depicted in Figure 2. AO samples were white in color throughout the heating process. By contrast, the color of AL samples changed from brown to white as the temperature increased from 500 °C to 700 °C, which indicates the decomposition of organic groups. The density and thermal conductivity at room temperature of all samples after annealing at 1000 °C are listed in Table 2. The increase in density and thermal conductivity of AO samples after heating at 1000 °C was smaller than for AL samples. AL7 and AL10, in particular, exhibited a high density and thermal conductivity after heat treatment at 1000 °C. Nevertheless, AO and AL samples exhibited broadly similar thermal stability, although AL4 had the lowest density (234 mg/cm^3^) and thermal conductivity (0.050 W/m∙K) after calcination at 1000 °C.

**Figure 3:**
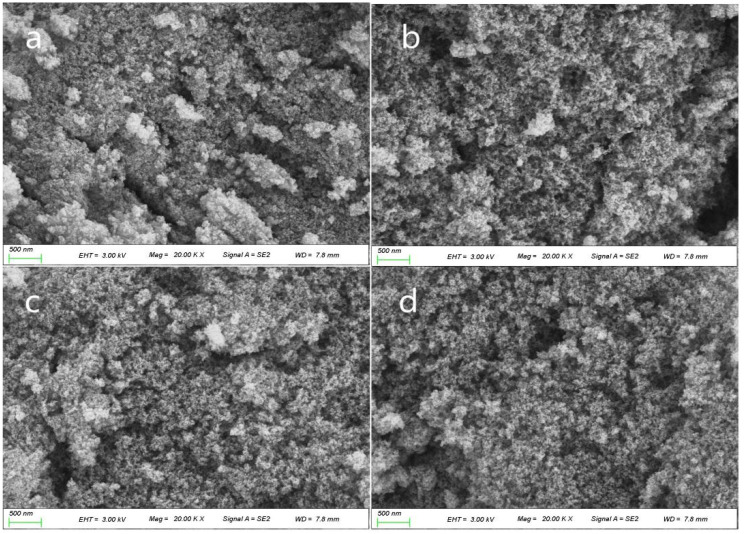
Scanning electron microscopy (SEM) images of sample AL7 after heat treatment from 300 to 900 °C: (**a**) AL7-300, (**b**) AL7-500, (**c**) AL7-700 and (**d**) AL7-900 (EHT: extra high tension, MAG: magnification, SE: secondary electrons, WD: work distance).

**Figure 4:**
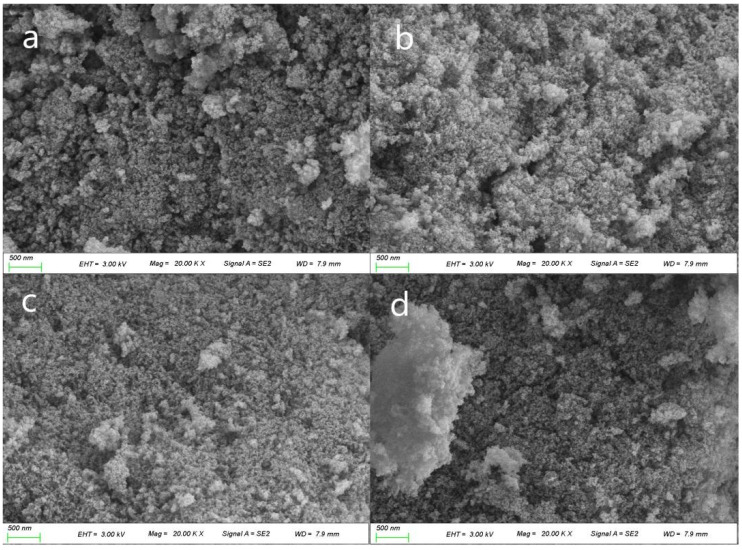
Scanning electron microscopy (SEM) images of AO7 sample after heat treatment from 300 to 900 °C: (**a**) AO7-300, (**b**) AO7-500, (**c**) AO7-700 and (**d**) AO7-900 (EHT: extra high tension, MAG: magnification, SE: secondary electrons, WD: work distance).

**Figure 5:**
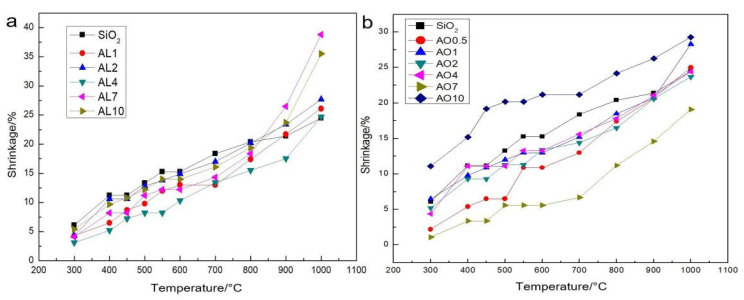
The linear shrinkage of (**a**) AL samples and (**b**) AO samples after heat treatments from 300 to 1000 °C (AL and AO represent the samples derived from AlCl_3_·6H_2_O and α-Al_2_O_3_ powders, respectively. The numbers denote the molar percentage of Al/(Al+Si)).

Figure 3 and Figure 4 exhibit the variation in the microstructures of samples AL7 and AO7 after heat treatment from 300 °C to 900 °C. Sample AL7 maintained a uniform nanoporous structure, and no apparent pore structure collapse was observed after high temperature calcination, which resulted in the low shrinkage shown in Figure 5. On the contrary, sample AO7 showed a comparatively denser pore structure that became denser and sintered as the calcination temperature increased. These results indicate that the AL sample possesses better thermal stability than the AO sample.

### 2.2. Shrinkage after Different Heating Temperatures

In order to analyze the thermal stability of samples, we focused on the linear shrinkage of all the samples after heat treatment from 300 to 1000 °C with an incremental step of 100 °C (Figure 5). As the temperature increased from 400 to 600 °C the incremental rise was 50 °C because of the potentially drastic change in aerogel structure over this temperature range. For AL samples, all alumina-doped silica aerogels exhibited lower linear shrinkage compared with pure silica aerogels after heat treatment before 800 °C. However, after heat treatment at 900 and 1000 °C, alumina-doped silica aerogels showed a large increase in linear shrinkage, especially samples AL7 and AL10. As the AL4 sample had the best thermal stability, it was selected for detailed study. Most AO samples exhibited lower linear shrinkage compared with pure silica aerogels. Moreover, there was no large increment of linear shrinkage from 900 to 1000 °C due to the stable crystal phase of α-Al_2_O_3_. By comparing the two figures, we found that AL samples exhibit lower linear shrinkage before calcination at 800 °C, while AO samples show lower linear shrinkage after calcination at 800 °C.

### 2.3. Variation of Chemical Composition at Different Temperatures

**Figure 6:**
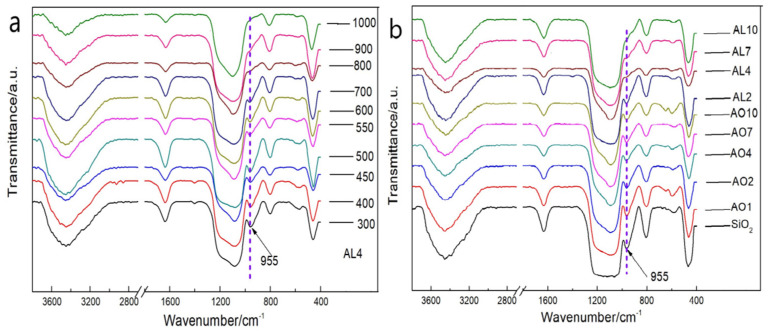
FTIR spectra of the (**a**) AL4 after heat treatment from 300 to 1000 °C, and FTIR spectra of the (**b**) AO and AL samples after heat treatment at 800 °C (AL and AO represent the samples derived from AlCl_3_·6H_2_O and α-Al_2_O_3_ powders, respectively. The numbers denote the molar percentage of Al/(Al+Si)).

Figure 6 shows the variation in chemical bonds of the AL4 and AO samples after heat treatment at different temperatures. With regard to the FTIR spectra of sample AL4 after heat treatment from 300 to 1000 °C, bands at 3450 and 1638 cm^−1^ indicate the presence of water adsorption [35]. The wide band at 1095 cm^−1^ corresponds to an antisymmetric stretching vibration peak of Si–O–Si, and the bands at 798 and 466 cm^−1^ correspond to a symmetric stretching vibration peak of Si–O–Si. These bands do not show significant changes with the increment of temperature. The bands at 2972, 2898 and 1402 cm^−1^ at under 400 °C indicate the Si–CH_3_ vibration of hydrocarbon groups and disappear after heat treatment at over 500 °C. The band at 955 cm^−1^, which is related to a bending vibration peak of Si–OH, decreases gradually with the increment of temperature and completely disappears after heat treatment at 800 °C. This result indicates a dehydration reaction of Si–OH that results in significant linear shrinkage. For the FTIR spectra of the AO and AL samples after heat treatment at 800 °C, the main peaks were related to water adsorption and the Si–O–Si bond, which are similar to the results for the AL4 sample. However, the band at 955 cm^−1^ relating to Si-OH reveals different conditions. The peak related to the Si–OH bond of AL samples almost disappears, except for in sample AL2, while the Si–OH bond of all the AO samples still existed after heat treatment at 800 °C, which resulted in a reduced linear shrinkage compared to all the AL samples. The Al–O–Al and AL–OH bonds at 880 and 1074 cm^−1^ [36,37] were not observed in the FTIR spectra due to the relatively rare doping content of alumina.

### 2.4. Crystal Phase of Aerogels after Heat Treatment at 800 °C

**Figure 7:**
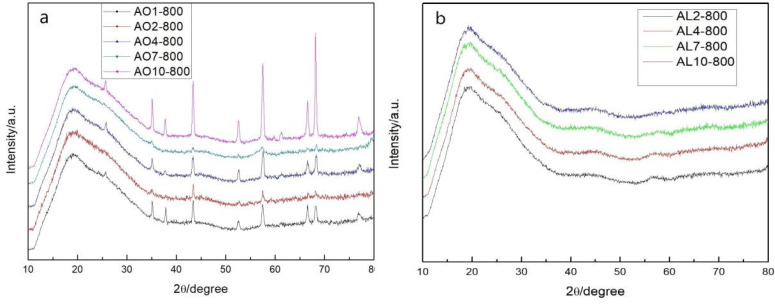
XRD patterns of (**a**) AO and (**b**) AL samples after heat treatment at 800 °C (AL and AO represent the samples derived from AlCl_3_·6H_2_O and α-Al_2_O_3_ powders, respectively. The numbers denote the molar percentage of Al/(Al+Si)).

Figure 7 shows the XRD pattern of AO and AL samples after heat treatment at 800 °C. All AO samples show diffraction peaks at 25.6°, 35.1°, 37.8°, 43.4°, 52.5°, 57.5°, 66.5° and 68.2°, which correspond to the α-Al_2_O_3_ phase (PDF 10–0173). This was expected because the AO samples were doped with α-Al_2_O_3_ powders. However, no AL samples exhibited a diffraction peak, indicating that the abundant silica content inhibited the phase transition of alumina.

### 2.5. SSA and Pore Size Distribution at Different Temperatures

**Figure 8:**
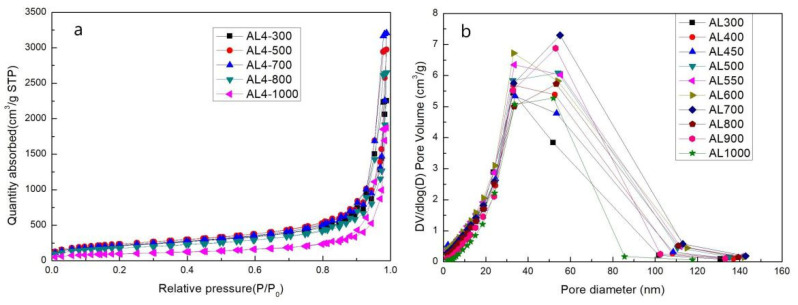
(**a**) Desorption isotherms and (**b**) pore size distribution of sample AL4 after heat treatment from 300 to 1000 °C.

**Figure 9:**
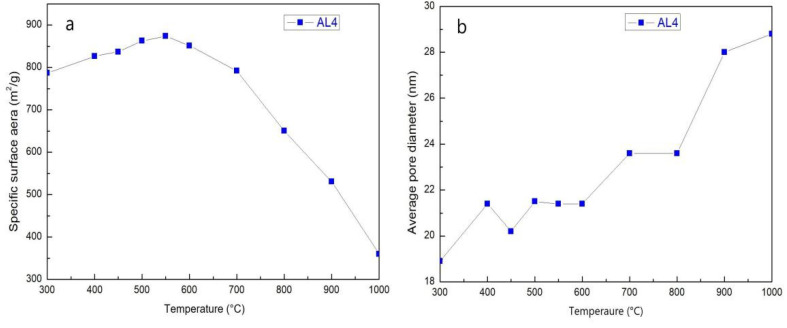
(**a**) Specific surface area (SSA) variation and (**b**) average pore diameter of sample AL4 with heat treatment from 300 to 1000 °C.

Figure 8 shows desorption isotherms and the pore size distribution of sample AL4. All samples exhibit the characteristic features of mesoporous materials (Type-IV isotherms). The pore structure and pore size distribution show nearly no change with the incremental increase in temperature from 300 to 1000 °C, which indicates high thermal stability. The specific data and variation tendencies are shown in Table 3 and Figure 9. It is obvious that the SSA of AL4 increases gradually before 550 °C and then decreases rapidly after 550 °C, which can be attributed to the decomposition of residual organic groups. This can be identified by the color change from brown to white of the AL4 sample after heating. The average pore diameter of AL4 increased generally, which indicates the irreversible collapse of the mesoporous structure. The change in SSA and average pore diameter was also relatively small before 800 °C. However, the SSA decreased significantly after heat treatment from 800 to 1000 °C. This result can be mainly attributed to a dehydration reaction of Si–OH.

**Figure 10:**
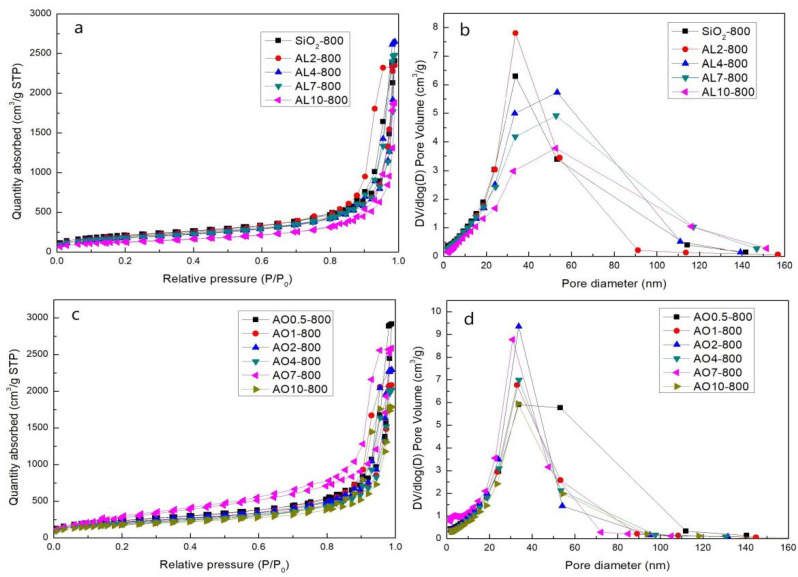
Desorption isotherms and pore size distribution of the (**a**,**b**) AL samples and (**c**,**d**) AO samples after heat treatment at 800 °C (AL and AO represent the samples derived from AlCl_3_·6H_2_O and α-Al_2_O_3_ powders, respectively. The numbers denote the molar percentage of Al/(Al+Si)).

Since the AL4 sample showed good thermal stability after being heat treated at 800 °C, we studied the properties of other samples after heat treatment at 800 °C. Figure 10 exhibits the Type-IV isotherms and mesoporous structure for all AO and AL samples. The pore diameter distributions of all samples show similar shapes, with only one peak, and the exact values are shown in Table 4. For AL samples, the SSA decreased gradually with the incremental rise of the proportion of alumina. The main reason for this is the dehydration reaction of Si–OH, as the hydrolysis of AlCl_3_·6H_2_O produces abundant bonds of Al–OH. For AO samples, the situation differed: The addition of α-Al_2_O_3_ powders reduced the dehydration reaction of Si–OH, increasing the SSA and decreasing the average pore diameter of AO samples. The AO7 sample, in particular, showed the highest SSA of 1056 m^2^/g, which is consistent with the lowest linear shrinkage of 11%.

### 2.6. Analysis of Sol-Gel Process and Thermal Stability

The sol-gel process is the key step when constructing a robust three-dimensional network of aerogels and can be divided into the hydrolysis and condensation stage. For AL samples during the hydrolysis stage, we adjusted the pH value to 1–2 by using HNO_3_. TEOS, ethanol, deionized water and AlCl_3_·6H_2_O reacted according to Equations (1) and (2) and formed abundant Si–OH and Al–OH bonds. During the condensation stage, we adjusted the pH value of the solution to 6–7 using NH_4_OH. Then, Si–OH and Al–OH bonds reacted according to Equations (3)–(5) and formed abundant Si–O–Si, Al–O–Al and Si–O–Al bonds. There were also residual –OH bonds, especially on the surface of aerogels, which react more at high temperatures. For AO samples, TEOS has the same reactions while α-Al_2_O_3_ powders are chemically stable. As the nanoparticles of α-Al_2_O_3_ powders have high surface activity, silica primary particles will aggregate on the surface of α-Al_2_O_3_ particles through van der Waals forces, the electrostatic force and hydrogen bonding [38].

The specific sol-gel mechanism can be described by Equations (1)–(5) [18].

(a) Hydrolysis.

Si(OC_2_H_5_)_4_ + *n*H_2_O --> Si(OH)*_n_*(OC_2_H_5_)_4−*n*_ + *n*C_2_H_5_OH (1)

Al_3_^+^ + 6H_2_O --> [Al(H_2_O)_6_]^3+^ --> [Al(OH)_n_(H_2_O)_6−n_]^(3−n)+^ + *n*H^+^ (2)

(b) Condensation.

–Si–OH + Si(OH)*_n_*(OC_2_H_5_)_4−*n*_ --> –Si–O–Si(OH)*_n_*(OC_2_H_5_)_4−*n*_ + H_2_O (3)

–Si–OH + [Al(OH)*_n_*(H_2_O)_6−*n*_]^(3−*n*)+^ --> [Si–O–Al(OH)*_n_*_−1_(H_2_O)_6-*n*_]^(3−*n*)+^ + H_2_O (4)

2[Al(OH)*_n_*(H_2_O)_6−*n*_]^(3−*n*)+^ --> [(H_2_O)_6−*n*_(OH)*_n_*_−1_Al–O–Al(OH)*_n_*_−1_(H_2_O)_6-*n*_]^2(3−*n*)+^ + H_2_O (5)

After heat treatment at a high temperature, AL samples have greater shrinkage than AO samples due to the dehydration condensation of –OH, which can be identified by the decrease in Si–OH bonds in the FTIR spectra and the decreased SSA. By contrast, AO samples exhibited better thermal stability due to their stable chemical properties and the crystal phase of α-Al_2_O_3_ particles. Thus, Si–OH bonds do not react completely at 800 °C and AO samples show less shrinkage and higher SSA.

## 3. Conclusions

In this work, we applied an acid-base two-step sol-gel method for the preparation of high-temperature heat-resisting alumina-doped silica aerogels using TEOS, AlCl_3_·6H_2_O and α-Al_2_O_3_ powders as the precursors. Alumina-doped silica aerogels were facilely prepared by two methods: Physical mixing and creating a chemical compound. Although both methods enhanced the thermal stability of silica aerogels before 800 °C, the hydrolysis of AlCl_3_·6H_2_O produces Al–OH and promotes the dehydration reaction of Si–OH. This gave the AL samples higher shrinkage and lower SSA after heat treatment at 800 °C. On the contrary, the addition of α-Al_2_O_3_ powders reduced the dehydration reaction of the silicon hydroxyl groups, and the 6.1 wt.% α-Al_2_O_3_ powders-doped sample exhibited the lowest linear shrinkage (11%) and highest SSA (1056 m^2^/g) after heat treatment at 800 °C for 1 h. The heat resistance is expected to be further improved through the composition with fiber in order to satisfy industrial requirements. This simple and rapid method can effectively enhance the thermal stability of silica aerogels and facilitate the large-scale production of low-cost thermal insulation applications of silica-based aerogels from 600 to 1000 °C.

## 4. Materials and Methods

### 4.1. Raw Materials

TEOS, aluminum chloride hexahydrate (AlCl_3_·6H_2_O), ethanol, nitric acid (HNO_3_, 68%), ammonia (NH_4_OH, 25%) and α-Al_2_O_3_ powders (with an average diameter of ≈30 nm) were purchased from Sinopharm Chemical Reagent Corporation (Shanghai, China). Deionized water was applied in all experiments. All reagents were analytical grade and were used as received without further purification.

### 4.2. Synthesis

TEOS (30 mL), ethanol (55 mL), and deionized water (5 mL) were added into a glass beaker to obtain solution A. Different proportions of AlCl_3_·6H_2_O or α-Al_2_O_3_ powders were dissolved or dispersed into ethanol in order to obtain solution B, which was added to solution A with vigorous stirring. Solution C comprised ethanol: HNO_3_ = 9:1, which was added to adjust the pH value of the solution to pH 1–2 with stirring for 20 min. Solution D consisted of ethanol: NH_4_OH = 9:1, which was added to adjust the solution to pH 6–7. After stirring for several minutes, the solution changed into a transparent gel with quiescence in room temperature for about 10 min. Samples with the molar percentage of Al/(Al+Si) = 1, 2, 4, 7, 10% were denoted as AL1, AL2, AL4, AL7 and AL10 for AlCl_3_·6H_2_O and AO1, AO2, AO4, AO7 and AO10 for α-Al_2_O_3_ powders, respectively.

The wet gels aged for one day and the solvent was displaced with fresh ethanol for 3 days. The ethanol supercritical drying was carried out in an autoclave heated to 260 °C and 10 MPa with a heating rate of 1 °C/min and was held for 1 h at 260 °C. The ethanol in the autoclave was then emitted gradually with a decompression rate of about 2 MPa/h. The obtained alumina-doped silica aerogels were heat-treated at 300, 400, 450, 500, 550, 600, 700, 800, 900 and 1000 °C and the corresponding samples were denoted by adding the heat treatment as a suffix (e.g., AL4-300 and AO1-800).

### 4.3. Characterization

The linear shrinkage was calculated depending on the change in the cylinder diameters of the aerogels with an increment in temperature. The samples compared were the as-prepared aerogels derived from super critical drying process without any treatment. Bulk density was determined by the mass and volume of regular cylinders. The surface morphology was observed by scanning electron microscopy (ZEISS Gemini 300, Oberkochen, Germany). The chemical groups that remained in the samples were investigated by a Fourier transform infrared spectrometer (FTIR, TENSOR27, Bruker, Karlsruhe, Germany), and all samples were dispersed in dry KBr and pressed into a semitransparent slice for FTIR characterization. The crystal phase of the aerogels was analyzed by powder X-ray diffraction (XRD) with a Rigata/max-C diffractometer using Cu-K_α_ radiation (Rigaku Ultima IV, Tokyo, Japan). The specific surface area (SSA) and pore size distributions were obtained by a N_2_ adsorption analyzer (ASAP2460). Thermal conductivity was measured by thermal constant analyzer (Hotdisk TPS 2500, Uppsala, Sweden).

## Figures and Tables

**Table 1:** Density and room-temperature thermal conductivity (measured by the hotdisk method) of different samples without heat treatment (AL and AO represent the samples derived from AlCl_3_∙6H_2_O and α-Al_2_O_3_ powders, respectively. The numbers denote the molar percentage of Al/(Al+Si)).

Sample	Density (mg/cm^3^)	Thermal Conductivity (W/m·K)	Sample	Density (mg/cm^3^)	Thermal Conductivity (W/m∙K)
AO0.5	151	0.0383 ± 0.0019	SiO_2_	132	0.0286 ± 0.0014
AO1	149	0.0374 ± 0.0020	AL1	134	0.0283 ± 0.0016
AO2	155	0.0340 ± 0.0017	AL2	140	0.0296 ± 0.0015
AO4	161	0.0360 ± 0.0019	AL4	114	0.0269 ± 0.0014
AO7	170	0.0357 ± 0.0018	AL7	102	0.0284 ± 0.0014
AO10	144	0.0304 ± 0.0015	AL10	103	0.0292 ± 0.0015

**Table 2:** Density and room-temperature thermal conductivity (measured by hotdisk method) of different samples after heat treatment at 1000 °C (AL and AO represent the samples derived from AlCl_3_·6H_2_O and α-Al_2_O_3_ powders, respectively. The numbers denote the molar percentage of Al/(Al+Si)).

Sample	Density (mg/cm^3^)	Thermal Conductivity (W/m∙K)	Sample	Density (mg/cm^3^)	Thermal Conductivity (W/m∙K)
AO1	273	0.058 ± 0.003	AL1	334	0.057 ± 0.003
AO2	277	0.054 ± 0.003	AL2	299	0.056 ± 0.003
AO4	315	0.056 ± 0.003	AL4	234	0.050 ± 0.003
AO7	280	0.055 ± 0.003	AL7	409	0.067 ± 0.003
AO10	314	0.056 ± 0.003	AL10	331	0.062 ± 0.003

**Table 3:** Specific surface area (SSA) and average pore diameter of sample AL4.

Sample	SSA (m^2^/g)	Pore Diameter (nm)	Pore Volume (cm^3^/g)
AL4-300	787 ± 3	18.9	3.5
AL4-400	827 ± 3	21.4	4.1
AL4-450	837 ± 3	20.2	3.9
AL4-500	862.8 ± 1.9	21.5	4.6
AL4-550	873.5 ± 1.3	21.4	4.8
AL4-600	851.5 ± 1.3	21.4	4.8
AL4-700	791.8 ± 1.2	23.6	5.0
AL4-800	650.3 ± 1.0	23.6	4.1
AL4-900	530.6 ± 0.8	28.0	4.2
AL4-1000	360.2 ± 0.6	28.8	2.9

**Table 4:** Specific surface area (SSA) and average pore diameter of AL and AO samples after heat treatment at 800 °C (AL and AO represent the samples derived from AlCl_3_·6H_2_O and α-Al_2_O_3_ powders, respectively. The numbers denote the molar percentage of Al/(Al+Si)).

Sample	SSA(m^2^/g)	Pore Dimeter (nm)	Pore Volumes (cm^3^/g)
AL2	683.9 ± 1.1	20.5	3.7
AL4	650.3 ± 1.0	23.6	4.1
AL7	652.5 ± 0.9	21.8	3.9
AL10	471.9 ± 0.7	22.2	2.9
AO0.5	871.9 ± 1.7	20.9	4.5
AO1	743.4 ± 1.7	17.8	3.3
AO2	786.0 ± 1.3	18.0	3.6
AO4	706.3 ± 1.3	17.9	3.2
AO7	1056 ± 12	12.2	4.0
AO10	633.0 ± 1.4	17.9	2.8

## Data Availability

The data presented in this study are available on request from the corresponding author.
